# Cardiovascular magnetic resonance 4D flow analysis has a higher diagnostic yield than Doppler echocardiography for detecting increased pulmonary artery pressure

**DOI:** 10.1186/s12880-020-00428-9

**Published:** 2020-03-06

**Authors:** Joao G. Ramos, Alexander Fyrdahl, Björn Wieslander, Gert Reiter, Ursula Reiter, Ning Jin, Eva Maret, Maria Eriksson, Kenneth Caidahl, Peder Sörensson, Andreas Sigfridsson, Martin Ugander

**Affiliations:** 1 Department of Clinical Physiology, Karolinska Institutet and Karolinska University Hospital, Stockholm, Sweden; 2 Siemens Healthcare Diagnostics GmbH, Graz, Austria; 3 Department of Radiology, Graz Medical University, Graz, Austria; 4 Siemens Medical Solutions, Cleveland, OH USA; 5 Department of Cardiology, Karolinska Institutet and Karolinska University Hospital, Stockholm, Sweden; 6 University of Sydney, Northern Clinical School, Sydney Medical School, Kolling Building, Level 12, Room, Sydney, 612017 Australia; 7 The Kolling Institute, Royal North Shore Hospital, St Leonards, Sydney, NSW 2065 Australia

**Keywords:** Magnetic resonance imaging, Pulmonary hypertension, 4D flow, Echocardiography

## Abstract

**Background:**

Pulmonary hypertension is definitively diagnosed by the measurement of mean pulmonary artery (PA) pressure (mPAP) using right heart catheterization. Cardiovascular magnetic resonance (CMR) four-dimensional (4D) flow analysis can estimate mPAP from blood flow vortex duration in the PA, with excellent results. Moreover, the peak systolic tricuspid regurgitation (TR) pressure gradient (TRPG) measured by Doppler echocardiography is commonly used in clinical routine to estimate systolic PA pressure. This study aimed to compare CMR and echocardiography with regards to quantitative and categorical agreement, and diagnostic yield for detecting increased PA pressure.

**Methods:**

Consecutive clinically referred patients (*n* = 60, median [interquartile range] age 60 [48–68] years, 33% female) underwent echocardiography and CMR at 1.5 T (*n* = 43) or 3 T (*n* = 17). PA vortex duration was used to estimate mPAP using a commercially available time-resolved multiple 2D slice phase contrast three-directional velocity encoded sequence covering the main PA. Transthoracic Doppler echocardiography was performed to measure TR and derive TRPG. Diagnostic yield was defined as the fraction of cases in which CMR or echocardiography detected an increased PA pressure, defined as vortex duration ≥15% of the cardiac cycle (mPAP ≥25 mmHg) or TR velocity > 2.8 m/s (TRPG > 31 mmHg).

**Results:**

Both CMR and echocardiography showed normal PA pressure in 39/60 (65%) patients and increased PA pressure in 9/60 (15%) patients, overall agreement in 48/60 (80%) patients, kappa 0.49 (95% confidence interval 0.27–0.71). CMR had a higher diagnostic yield for detecting increased PA pressure compared to echocardiography (21/60 (35%) vs 9/60 (15%), *p* < 0.001). In cases with both an observable PA vortex and measurable TR velocity (34/60, 56%), TRPG was correlated with mPAP (R^2^ = 0.65, *p* < 0.001).

**Conclusions:**

There is good quantitative and fair categorical agreement between estimated mPAP from CMR and TRPG from echocardiography. CMR has higher diagnostic yield for detecting increased PA pressure compared to echocardiography, potentially due to a lower sensitivity of echocardiography in detecting increased PA pressure compared to CMR, related to limitations in the ability to adequately visualize and measure the TR jet by echocardiography. Future comparison between echocardiography, CMR and invasive measurements are justified to definitively confirm these findings.

## Background

Pulmonary hypertension is defined as a mean pulmonary artery (PA) pressure (mPAP) equal to or greater than 25 mmHg assessed invasively by right heart catheterization (RHC) [1]. It affects approximately 1% of adults and is associated with high morbidity and mortality [2].

In clinical routine, pulmonary artery pressure is screened for using Doppler echocardiography [3] by measuring peak systolic tricuspid regurgitant jet velocity (TR) and deriving the peak systolic tricuspid regurgitant pressure gradient (TRPG). Furthermore, mPAP can also be estimated with echocardiography by adding mean right atrial pressure to TRPG, and a calibration factor [4]. However, these parameters tend to over- or underestimate pulmonary pressure compared with invasive measurements [5, 6]. Notably, the usefulness of echocardiography for follow-up and monitoring of treatment in pulmonary hypertension has been shown to be limited [7].

Cardiovascular magnetic resonance (CMR) has been used to estimate mPAP and diagnose pulmonary hypertension [8, 9]. Specifically, the duration, expressed as percentage of the cardiac cycle, of blood flow vortices in the pulmonary trunk assessed by CMR four-dimensional (4D) flow analysis has shown excellent correlation with invasively measured mPAP in previous studies [8, 10, 11]. This method yielded accurate results in all five world health organization (WHO) groups of pulmonary hypertension with high diagnostic sensitivity and specificity [11].

Estimation of mPAP by CMR has not yet been adapted in widespread clinical use, yet it is of great clinical interest to non-invasively, accurately, and precisely estimate PA pressure for the purposes of screening, diagnosis, prognosis, and for monitoring the effects of therapy. However, CMR mPAP and echocardiography TRPG have not yet been compared head-to-head. While TRPG and mPAP are not directly comparable from a physiological standpoint, in practice, TRPG is the main estimator of PA pressure in a clinical setting and is routinely used for screening of PH.

Therefore, the aim of this study was to compare agreement and diagnostic yield for evaluation of pulmonary hypertension between CMR and echocardiography in a clinical consecutive patient population.

## Methods

### Study participants

In this prospective study, we included 60 consecutive patients referred for a clinical CMR exam, who also had undergone or were scheduled for a clinically motivated transthoracic echocardiography. Patients with no contraindications for CMR were considered for inclusion if there was no atrial fibrillation and if the difference between exam dates was less than 60 days. Studies with poor image quality were excluded (*n* = 2). Approval was obtained from the appropriate local ethical committee and all participants provided written informed consent.

### Pre-CMR screening

Pre-CMR screening of all patients included a standard 12-lead electrocardiogram (ECG), blood pressure measurement and a brief history to rule out contraindications to CMR, as per clinical routine. All ECGs were obtained on a GE Marquette system (GE, Little Chalfont, United Kingdom).

### CMR acquisition

All CMR images were obtained either at 1.5 T (*n* = 43) or 3 T (*n* = 17) (MAGNETOM Aera or MAGNETOM Skyra, Siemens Healthcare, Erlangen, Germany) with ECG gating and phased array receiver coils.

Flow data in the pulmonary artery were acquired with 6–10 gapless slices using a retrospectively electrocardiographically gated two-dimensional spoiled-gradient-echo-based cine phase contrast sequence, with velocity encoding of 90 cm/s in all three spatial directions and three-fold averaging to suppress breathing artifacts. There was no navigator compensation. Typical image acquisition parameters were field of view 340 × 276 mm^2^, matrix 192 × 112 pixels, slice thickness 6 mm, bandwidth 449 Hz/Px, generalized autocalibrating partial parallel acquisition (GRAPPA) factor 2, autocalibration signal (ACS) 22 lines, flip angle 15°, TR/TE 6.41/4.10 ms, temporal resolution 77 ms interpolated to 20 cardiac phases per cardiac cycle, total imaging duration 6–11 min depending on heart rate and number of slices necessary to cover the main PA in an approximately sagittal orientation [10].

Additionally, the protocol included 2-, 3- and 4-chamber balanced steady-state free precession (bSSFP) cine images of the left ventricle as well as a complete short axis (SA) stack. Typical image parameters included field of view 380 × 320 mm^2^, matrix size 256 × 143 pixels with 1.5 × 1.5 mm^2^ in-plane resolution slice thickness 6 mm, bandwidth 930 Hz/Px, TR/TE 2.78/1.16 ms, temporal resolution 36 ms interpolated to 35 cardiac phases per cardiac cycle.

### CMR analysis

**Fig. 1:**
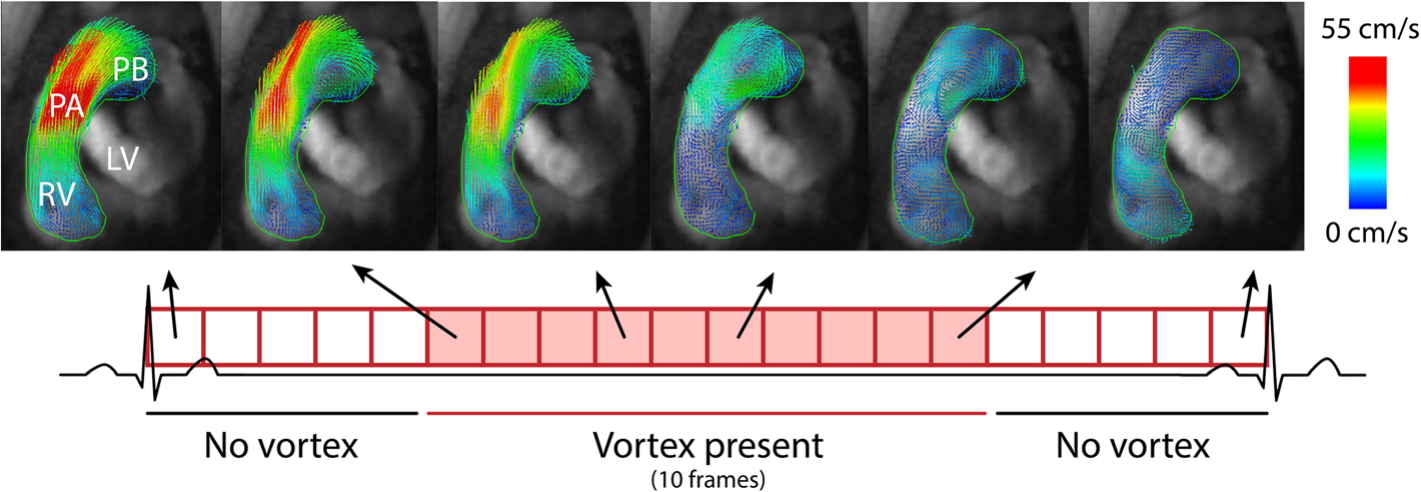
Schematic representation of the CMR 4D flow analysis method for estimation of PA pressure. Each of the boxes with red outline represent a single image from one time frame in the cardiac cycle. The boxes shaded red represent the time frames in which a vortex could be visualized. The top panels show a 3D vortex visualization of pulmonary flow in the right ventricle outflow tract orientation in a representative patient, with the black arrows denoting their location in the cardiac cycle. Visualization was performed using vector arrows, and the color scale denotes velocity of the respective vectors. RV – right ventricle, LV – left ventricle, PA – main pulmonary artery, PB – pulmonary bifurcation

Left ventricular volumes, mass and ejection fraction (LVEF) were measured from the short-axis cine stack using manual delineations in the software Siemens syngo. Via 4.1 (Siemens Healthcare, Erlangen, Germany). 4D Flow analysis was performed using prototype software (4D Flow, Siemens Healthcare, Erlangen, Germany), blinded to the results of echocardiography. After fully automated eddy current compensation and phase unwrapping, images were manually segmented in order to seed pixels in the right ventricle outflow tract and pulmonary artery only. Streamline visualization was used to visualize the flow in the right ventricular outflow tract. Next, 3D vector visualization was used to detect a blood flow vortex in the main pulmonary artery as previously described [8, 10]. Vortex assessment was performed by two trained observers (JGR and BW). Vortex duration in percentage of the cardiac cycle was calculated based on the ratio of frames with a visible concentric vortex, and the total number of frames. This process is illustrated in Fig. 1. Mean pulmonary arterial pressure (mPAP) was then estimated using the previously described empirically determined equation, $mPA{P}_{CMR}(mmHg)=\frac{Vortex\ duration\ \left(\%\right)+25.44}{1.59}$ [11]. Increased PA pressure by CMR was defined as a vortex duration ≥15% of the cardiac cycle, corresponding to an estimated mPAP ≥25 mmHg. CMR 4D flow analysis divided patients in three groups: (1) no visible vortex (duration 0%, normal pressure assumed), (2) vortex duration under 15% (normal pressure) and (3) vortex duration greater than or equal to 15% (elevated pressure).

### Echocardiography

Comprehensive transthoracic echocardiography was performed on all patients, including Doppler measurements of TR, using a commercially available system (Epiq, Philips, Amsterdam, Netherlands). Recordings were obtained from views including the left parasternal modified RV long axis, left parasternal short axis, and the apical four chamber views. All results were calculated as mean of three consecutive TR velocities, as measured from the view from where the Doppler TR jet was maximal and best defined. TRPG in mmHg was obtained from TR velocity using the equation *TRPG* = 4 *velocity* (*m*/*s*)^2^. Increased PA pressure by echocardiography was defined as TR jet velocity > 2.8 m/s, corresponding to an estimated TRPG > 31 mmHg. Echocardiographic mPAP was calculated using the Chemla equation, *mPAP* = 0.61 *sPAP* (*mmHg*) + 2 [12].

Similar to CMR, echocardiography patients were divided into three groups: (1) TR not measurable (normal pressure assumed), (2) TR velocity less than or equal to 2.8 m/s (normal pressure) and (3) TR velocity greater than 2.8 m/s (increased pressure).

### Statistics

Statistical testing was performed using freely available software (RStudio 2.1, Boston, MA, USA). Continuous variables were reported as mean ± standard deviation if normally distributed according to the Kolmogorov-Smirnov test, or median [interquartile range] as appropriate. Categorical variables were presented as percentages. To assess interobserver agreement for determination of vortex duration, we calculated bias, interobserver variability, and the interclass correlation of the vortex duration measurements of both readers. Comparison between echocardiographic and CMR measurements was performed using linear regression and Bland-Altman analysis. Categorical agreement between CMR and echocardiography with regards to detecting an increased PA pressure was performed with Cohen’s kappa. Diagnostic yield was defined as the fraction of participants with positive findings [13]. Differences in diagnostic yield were tested using McNemar’s exact test. A *p*-value less than 0.05 was considered statistically significant.

## Results

### Study participants

The study included consecutive patients (*n* = 60) referred for CMR due to known or suspected cardiopulmonary disease. Time between echocardiography and CMR was 6 [1–20] days. Patient characteristics are summarized in Table 1 and CMR characteristics are summarized in Table 2. Data on clinical characteristics were acquired at the time of CMR. When comparing the time points of CMR and echocardiography, there were no significant differences in HR (*p* = 0.07), systolic pressure (*p* = 0.91) or diastolic pressure (*p* = 0.06).

**Table 1:** Patient demographics and clinical characteristics

Number of patients, n	60
Age, years	60 [48–68]
Female, n (%)	20 (33)
Height, cm	174 ± 9
Weight, kg	79 ± 15
BMI, kg/m^2^	26 ± 4
BSA, m^2^	1.95 ± 0.23
Systolic blood pressure, mmHg	127 ± 20
Diastolic blood pressure, mmHg	74 ± 9
Heart rate, beats per minute	65 [59–81]
Smoking	
Never smoked, n (%)	40 (67)
Former smoker, n (%)	16 (27)
Current smoker, n (%)	4 (7)
Drugs	
Beta-blocker, n (%)	40 (67)
Angiotensin receptor blocker, n (%)	9 (15)
Calcium channel blocker, n (%)	10 (17)
Diuretic, n (%)	17 (28)
Angiotensin converting enzyme inhibitor, n (%)	16 (28)
Ticagrelor or clopidogrel, n (%)	17 (29)
Warfarin, n (%)	5 (8)
Diagnosis by CMR	
Ischemic myocardial disease, n (%)	18 (30)
Acute myocardial infarction, n (%)	11 (18)
Dilated cardiomyopathy, n (%)	6 (10)
Hypertrophic cardiomyopathy, n (%)	5 (8)
Amyloidosis, n (%)	2 (3)
Myocarditis, n (%)	3 (5)
Pericarditis, n (%)	3 (5)
Normal findings, n (%)	3 (5)
Other, n (%)	6 (10)
NYHA class	
0	38 (63)
1	12 (20)
2	5 (8)
3	5 (8)
4	0 (0)

Continuous variables reported as mean ± standard deviation if normally distributed, otherwise reported as median [interquartile range]. Categorical variables reported as n (% of total)

**Table 2:** CMR characteristics

Left ventricular end diastolic volume, ml	175 [134–207]
Left ventricular end diastolic volume index, ml/m^2^	56 [73–104]
Left ventricular end systolic volume, ml	76 [59–97]
Left ventricular end systolic volume index, ml/m^2^	40 [31–51]
Left ventricular mass, g	132 [113–157]
Left ventricular mass index, g/m^2^	68 [60–80]
Left ventricular ejection fraction, %	56 [48–62]
Vortex duration, % of cardiac cycle	10 [0–20]
Estimated mean pulmonary artery pressure, mmHg	22 [16–29]

Variables reported as median [interquartile range]

### Inter-observer variability on duration of vortical blood flow

We quantified interobserver variability using the absolute values of vortex duration as a percentage of the cardiac cycle. Mean values of vortex duration in the pulmonary artery were 9.5 ± 9.7% corresponding to an mPAP of 22.0 ± 6.1 mmHg for reader 1 and 10.8 ± 9.8% corresponding to an mPAP of 22.8 ± 6.2 mmHg for reader 2, with an average measurement between both readers of 10.1 ± 9.4% corresponding to an mPAP of 22.4 ± 5.9 mmHg. Interobserver variability was 3.9%, corresponding to an mPAP of 2.5 mmHg. There was minimal bias between readers (95% CI: 1.4–3.9%, and 0.9–2.5 mmHg). The intraclass correlation coefficient between measurements was 0.85.

### Detection of elevated pulmonary arterial pressure with CMR vs echocardiography

The frequency and proportion of patients in each diagnostic category are summarized in Table 3. Among the patients who had increased CMR mPAP but normal TRPG by echocardiography, TRPG was 27 [20–30] mmHg.

**Table 3:**
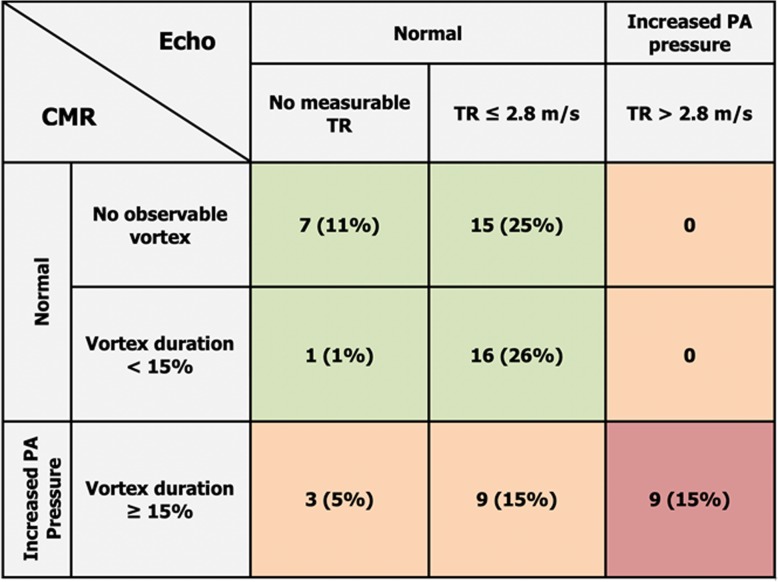
Frequency of patients in each diagnostic group when comparing cardiovascular magnetic resonance (CMR) and echocardiography (Echo). TR denotes tricuspid regurgitant jet velocity

### Comparison between estimated mPAP by CMR 4D flow analysis and TRPG by echocardiography

**Fig. 2:**
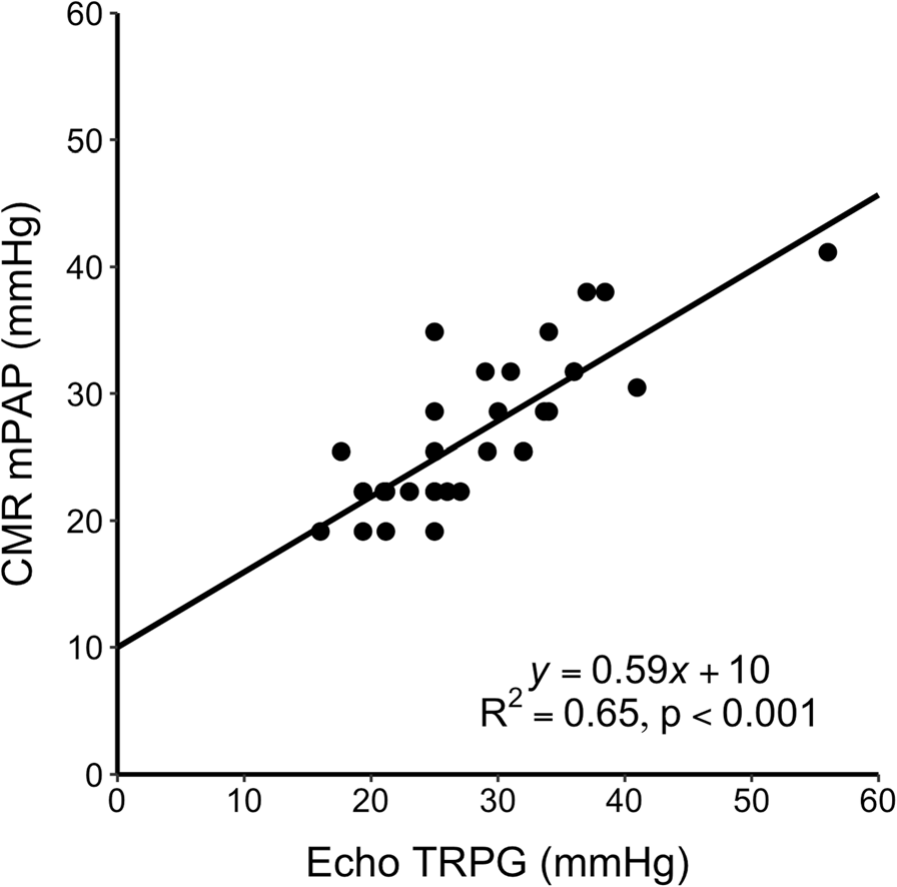
Linear regression (solid line) of estimated mean pulmonary artery pressure (mPAP) by cardiovascular magnetic resonance (CMR) and the tricuspid regurgitation (TR) pressure gradient (TRPG) by echocardiography (Echo) in patients with both observable vortex and measurable TR

For those patients that had both a detectable vortex in the pulmonary artery and a measurable TR (*n* = 34/60, 56%), comparisons of pulmonary artery pressure estimates are shown in Fig. 2. Linear regression yielded an R^2^ = 0.65, *p* < 0.001.

**Fig. 3:**
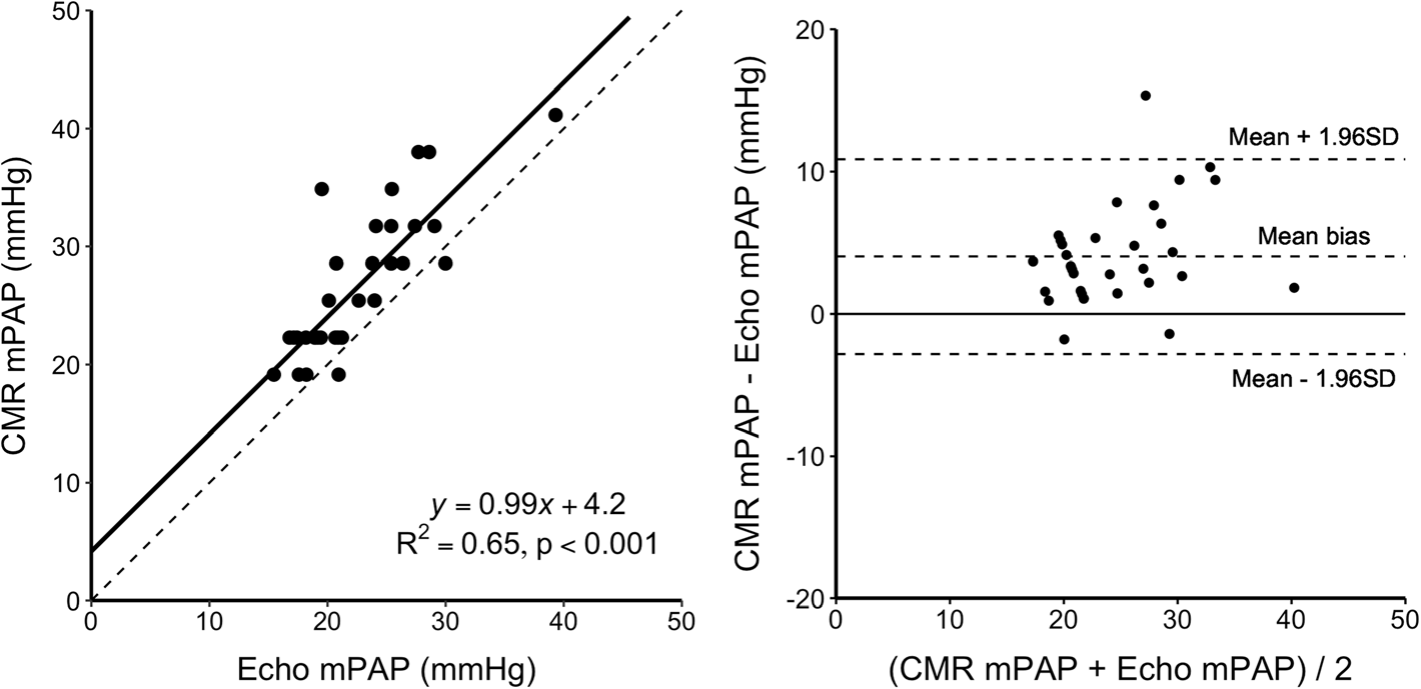
Left panel: Linear regression (solid line) of estimated mean pulmonary artery pressure (mPAP) by cardiovascular magnetic resonance (CMR) and estimated mPAP from TRPG by echocardiography (Echo). Dotted line shows line of identity. Right panel: Bland-Altman plot of estimated mPAP by CMR and estimated mPAP from TRPG by echocardiography in patients with both observable vortex and measurable TR. Mean bias 4.0 ± 6.9 mmHg

A comparison between CMR mPAP and echocardiography mPAP derived from TRPG is shown in Fig. 3. Linear regression yielded R^2^ = 0.65, *p* < 0.001. Mean difference between CMR mPAP and echocardiography mPAP estimated from TRPG was 4.0 ± 6.9 mmHg, assuming a right atrial pressure of 5 mmHg.

### Comparison between diagnostic yield of CMR 4D flow analysis and echocardiography in PH diagnosis

**Fig. 4:**
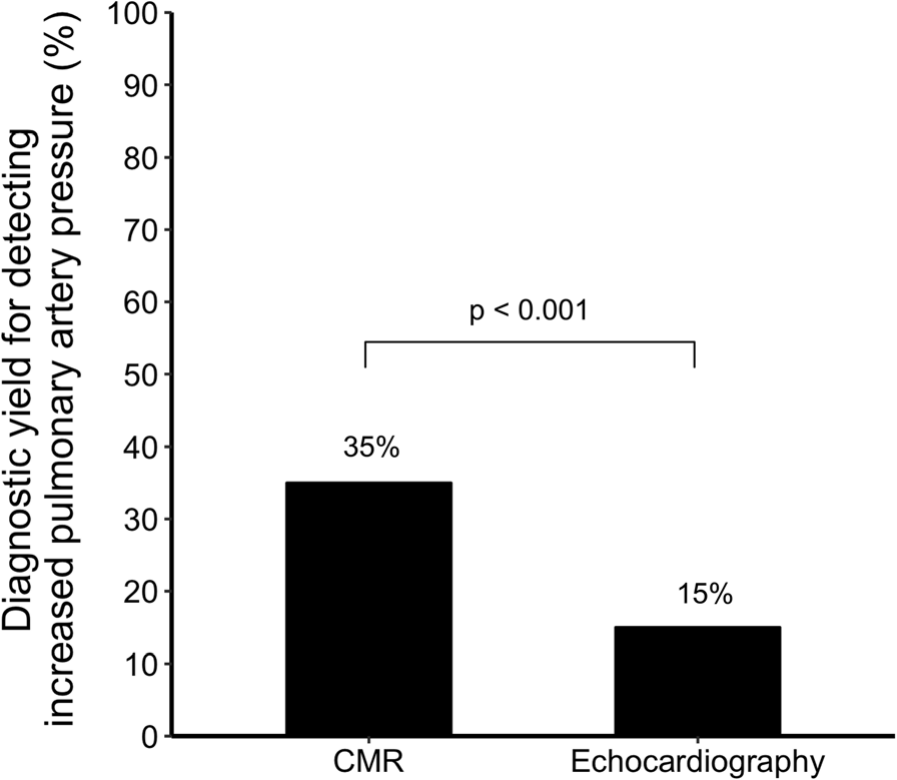
Diagnostic yield for detecting increased pulmonary artery pressure by either a 4D flow pulmonary artery vortex duration ≥15% of the cardiac cycle by cardiovascular magnetic resonance (CMR), or a tricuspid regurgitation jet velocity > 2.8 m/s by transthoracic Doppler echocardiography in all patients (*n* = 60)

There were 21 patients with increased PA pressure by CMR, and 9 patients with increased PA pressure by echocardiography. This translated into a higher diagnostic yield for detecting increased PA pressure by CMR compared to echocardiography (21/60 (35%) vs 9/60 (15%), *p* < 0.001). Figure 4 shows the difference in diagnostic yield between both methods. Cohen’s kappa for categorical agreement was 0.49 (95% confidence interval: 0.27–0.71).

## Discussion

The major finding of this study is that while there is a good quantitative and fair categorical agreement between CMR and echocardiography with regards to detecting and quantifying increased PA pressure, CMR had a more than twice as high diagnostic yield for detecting increased PA pressure.

The method used to estimate mPAP with CMR has been previously described and validated compared to RHC [8]. It was applicable in all types of PH, including acquired pulmonary arterial hypertension, with very good results compared to invasive measurements [11]. We decided to closely replicate the vortex method as originally described, with a time-resolved 2D acquisition of flow data in three spatial directions, which are then analyzed in 4D flow software. While this approach is not what is commonly known as 4D Flow CMR, the method measures time-resolved three-directional blood flow velocities in a volume, and did show an excellently reproducible ability to estimate PA pressures. In these studies, CMR mPAP was compared to RHC mPAP in all clinical subtypes of pulmonary hypertension. Our study is therefore the first to implement and test this method in an independent center with good results. When both methods yielded detectable estimates of PA pressure, we found good agreement between CMR mPAP and echo TRPG, which supports the accuracy of blood flow vortex duration in mPAP estimation. Interestingly, our regression equation when comparing CMR mPAP with TRPG was remarkably similar to Chemla’s equation to convert TRPG to mPAP, albeit with an assumed right atrial pressure of 5 mmHg. Furthermore, since echocardiography *mPAP* = 0.61 *sPAP* + 2 (Equation 1) [12] and *sPAP* = 4*v*^2^ + *RAP* (Equation 2), by assuming a RAP of 5 mmHg [14], by substitution we get

3 $$ mPAP=0.61\  TRPG+5 $$

which is the relationship one would mathematically expect between mPAP and TRPG. Interestingly, this derived equation is very similar to the regression equation we obtained in Fig. 2, i.e. *y* = 0.59 *x* + 10.

Our results show a low mean difference of 4.0 ± 6.9 mmHg between CMR mPAP and echocardiographic mPAP calculated using Eq. 3. This result assumes an estimated mean right atrial pressure of 5 mmHg, which is within the normal range. We did not use right atrial pressure estimates from echocardiography, since its estimation yields poorer agreement, with an accuracy as low as 34% [15]. Moreover, assuming a right atrial pressure of 3, 8 or 15 mmHg did not change the results in any meaningful way, with a mean bias consistently below 5 mmHg.

In 20% of cases, patients that had a normal or non-measurable TR also had a vortex duration indicating increased PA pressure by CMR. This group of patients did not have any identifiable characteristics that differed from the remaining patient cohort. Indeed, patients in this group were hemodynamically stable and diverse in terms of underlying diagnosis, none of them being disproportionately represented. By comparison, there were no patients with normal vortex duration that had an increased TR velocity.

Other reasons for the discrepancy between CMR and echocardiography could be a need for different thresholds for either CMR mPAP or echocardiographic TR velocity, or methodological limitations inherent to CMR or echocardiography with regards to the ability to either detect vortex presence, or accurately measure the peak TR velocity due to the angle of the main direction of Doppler flow. Using the current dataset, it is not possible to discern which of these is the main source of the discrepancy between CMR and echocardiography, and future studies are justified to address this question.

Some authors have challenged the accuracy of echocardiography when compared to invasive measurements, especially with regard to the utility of serial measurements [16, 17]. The REVEAL study, which compared peak systolic PA pressure between echocardiography and RHC, showed that in 44% of patients, there was a discordance in the estimation of PA pressure of at least 20% [5]. By comparison, mPAP estimation by CMR has shown excellent agreement with invasive RHC (R^2^ = 0.95) and excellent precision (standard deviation of the difference between CMR estimation and RHC measurement of mPAP 3.9 mmHg) [11].

CMR more than doubled the diagnostic yield of PA pressure estimation compared to echocardiography (35% vs 15%). Since TR is not always present nor measurable in all patients [18] and CMR 4D flow analysis has shown very good agreement with RHC in previous studies, it is plausible that CMR detects increased PA pressure in patients with undetectable TR. Future studies are needed to confirm this, and in such studies it would be of value to include both CMR, echocardiography and RHC measurements for simultaneous comparison of all three modalities.

### Limitations

The current study had some important limitations. First, echocardiography was not performed immediately after CMR, with a difference between exam dates of maximally 53 days and a median difference of 6 days, with 75% of patients having a difference of less than 20 days between exams. Despite the possibility of hemodynamic changes or relevant clinical events, other similar comparative studies with echocardiography have allowed for even longer periods with no significant impact of time difference between exam dates [5]. It would also be beneficial to guarantee inclusion of all clinical types of PH, to confirm the effectiveness of the vortex duration method in all hemodynamic circumstances. Lastly, data from RHC would be necessary to definitively establish a potential superiority of this CMR method compared with echocardiography. While diagnostic yield is higher in CMR, it is not possible to claim that the diagnosis of elevated PA pressure was correct in all CMR cases without invasive measurements. Despite this, our results show that there is a notable discrepancy between CMR and echocardiography with regards to detection of increased PA pressure at the existing clinical thresholds.

### Future directions

Estimation of mPAP with CMR is not currently performed routinely in the clinical setting. However, there are several hemodynamic parameters relevant to pulmonary hypertension that can be assessed by CMR [19]. Further validation of these techniques may warrant their introduction in clinical protocols, as they allow a more comprehensive physiologic evaluation of the heart. In particular, CMR may become an alternative to repeated RHC mPAP measurements for follow-up in pulmonary hypertension, provided that these results are can be confirmed by invasive measurements in the future.

## Conclusions

There is good quantitative and fair categorical agreement between estimated mPAP from CMR and TRPG from echocardiography. CMR more than doubles the diagnostic yield for detecting increased PA pressure compared to echocardiography. This is potentially due to a lower sensitivity of echocardiography in detecting increased PA pressure compared to CMR, possibly related to limitations in the ability to adequately visualize and measure the TR jet by echocardiography. Comparison with invasive measurements is warranted in order to confirm these results.

## Data Availability

The datasets used and analyzed during the current study are available from the corresponding author on reasonable request.

## Abbreviations

CMR: Cardiovascular magnetic resonance
mPAP: Mean pulmonary artery pressure
PA: Pulmonary artery
RHC: Right heart catheterization
TR: Peak tricuspid regurgitation velocity
TRPG: Tricuspid regurgitation pressure gradient
